# Interest of Thin Skin Flaps in the Treatment of Postburn Cervical Contractures: About Five Cases

**DOI:** 10.1155/2019/9879163

**Published:** 2019-05-19

**Authors:** I. Ghorbel, F. Bouaziz, H. Bellaaj, S. Moaalla, Kh. Ennouri

**Affiliations:** ^1^Department of Plastic and Aesthetic Surgery, Habib Bourguiba University Hospital, Rue el Ferdous 3029 Sfax, Tunisia; ^2^University of Sfax, Faculty of Medicine, Avenue Majida Boulila, 3029 Sfax, Tunisia

## Abstract

**Objectives:**

The use of thin skin flaps in the reconstruction of postburn cervical contractures associated with functional impacts.

**Material and Methods:**

We conducted a descriptive observational study on about five patients who had reconstruction of postburn cervical contractures using various thin skin flaps in a plastic surgery department. The follow-up period was ranged from 12 to 18 months. We determined the characteristics of the retraction according to the Vandenbussche classification, the indications of the flaps, and the functional and aesthetic results.

**Results:**

Most of our patients were young adults presenting segmental retraction of the neck associated with moderate functional impact. The occipito-cervico-dorsal flap was used in three cases. The occipito-cervico-shoulder flap was used in two cases. The aesthetic and functional results were satisfactory for all patients, but a “neck-collar” aspect of the cervical-chin angle was noticed.

**Conclusion:**

Postburn cervical contractures remain frequent. Their reconstruction is a difficult process as it is ideally done with a skin similar to the original one. The use of thin skin flaps seems to be an excellent option.

## 1. Introduction

Postburn cervical contractures remain frequent in our country. Their reconstruction is ideally done with a skin having similar mechanical and aesthetic qualities as the original one. It is aimed at restoring the cervical-chin angle (CCA), the cutaneous elasticity, and the thoracic component [1]. The choice of the reconstructive procedure depends on the type and the size of retraction. Skin grafts are often complicated by dyschromia, hypertrophy, and/or a keloid scar [1–3]. Local random flaps have a limited surface area and often require prior iterative expansion [1, 4]. The free flaps are rarely used in our practice because of the bulky skin poorly adapted to the cervical region, the long-time procedure, and the nonavailability of adequate material in our operation room [1, 5]. Three variants of the occipito-cervico flap were described, based on the descending cutaneous branches of the occipital artery [2]. They are the occipito-cervico-dorsal (OCD) flap, occipito-cervico-shoulder (OCS) flap, and occipito-cervico-pectoral (OCP) flap. These flaps bring skin having similar qualities as the skin of the neck. They allow the elevation of large skin pallet [2, 3, 6–9]. They have been called “superthin flaps” or thin skin flaps [4, 10]. They gather the thinness of the skin grafts with the vascular autonomy of the flaps. However, their advantages over other locoregional flaps such as the supraclavicular flap [11] and the deltopectoral flap [12] have not been well established. Our purpose is to evaluate the place of these flaps in the reconstruction of postburn cervical contractures.

## 2. Material and Methods

**Figure 1:**
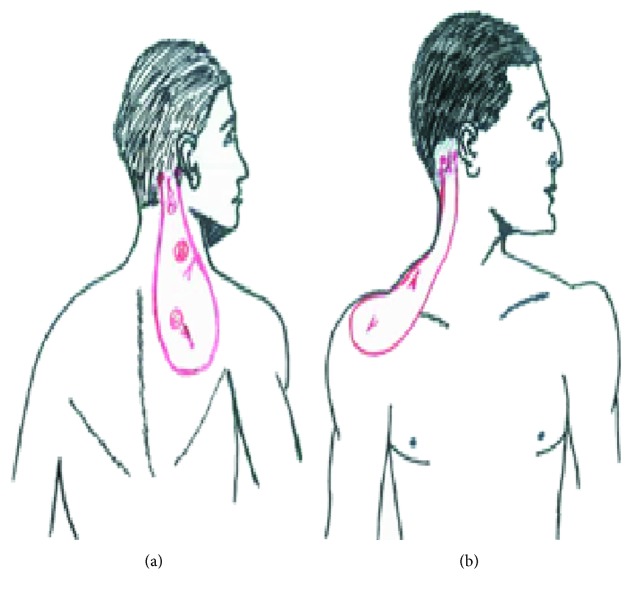
Types of occipito-cervico flaps used in our cases: (a) occipito-cervico-dorsal flap and (b) occipito-cervico-shoulder flap.

We conducted a descriptive observational study ranging from 2013 to 2016. Five patients had thin skin flaps to repair postburn cervical contractures in our Department of Plastic, Reconstructive, and Aesthetic Surgery. Two variants of occipito-cervico flaps were used: the occipito-cervico-dorsal (OCD) flap and the occipito-cervico-shoulder (OCS) flap (Figures 1(a) and 1(b)). This later was preceded by an expansion. The expander was inserted under the skin of the supraclavicular region. Approximately 180 cc of saline was injected during two months with a rhythm of one injection per two weeks.

We determined the characteristics of the retraction according to the Vandenbussche classification [13], the indication of the occipito-cervico flaps, the functional outcome, and the aesthetic result of both donor and recipient sites.

## 3. Result

There were four men and one woman in our series. Their ages ranged from 14 to 37 years old. All patients had thermal burns. Segmental retraction with a minimal functional repercussion was noticed in four cases. Only one patient had a major retraction. Depending on the type of the flap used, our patients were divided into 2 groups.

**Figure 2:**
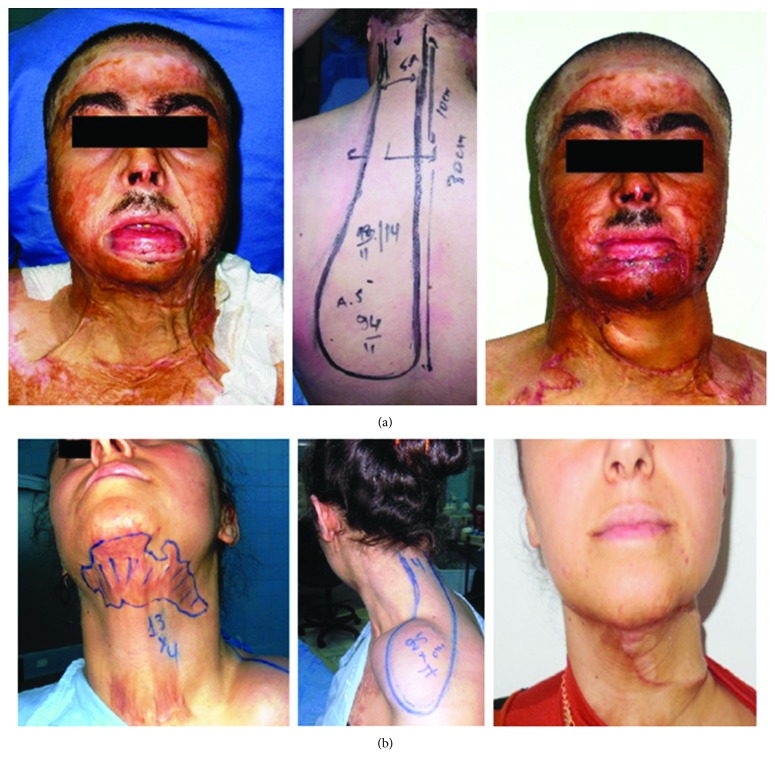
(a) Man, 29 years old, complete retraction with major impact on the extension of the neck, occipito-cervico-dorsal flap design, transposition to the cervical region, preliminary result. (b) Woman, 25 years old, minor retraction of the neck, occipito-cervico-shoulder flap design, transposition to the cervical region, preliminary result.

The occipito-cervico-dorsal (OCD) flap was practiced for three patients, all males (Table 1). In the first case, the patient had complete retraction of the neck: the cervical-chin angle (CCA) disappeared totally, and a thoracic retraction was observed. There was also a major impact on the extension of the neck. In the remaining two cases, the patients had a band scar on the lateral side of the neck covering its horizontal and vertical parts, with minimal limitation of extension. The flap was designed in a racquet shape (Figure 1(a)). The width of the cutaneous pedicle in the nuchal region measured 4-5 cm. The size of the flap varied between 24 and 30 cm in length and between 7 and 10 cm in width. The thinning of the skin flap was performed in two cases with no impact on the vitality of the skin pallet. In all cases, the flap was transposed to the cervical region at the angle of 110 degrees. The donor site was closed as a first intention. A distal venous congestion occurred within the first days after surgery and was improved in all cases. Total healing was obtained between 29 and 36 days. The follow-up ranged from 12 months to 24 months. The functional result was considered satisfactory in all cases with complete neck mobility and good skin quality. However, the cervical-chin angle was not restored with a “neck-collar” appearance of the flap (Figure 2(a)). For the donor site, the scar was large and unsightly with persistence of a cutaneous “dog ear” at the base of the flap (Figure 2(a)) requiring surgical revision in two cases.

The occipito-cervico-shoulder (OCS) flap was used in two patients, a woman and a male child (Table 2). A medial or lateral-cervical scar fold involving the horizontal part of the neck was noticed in both cases with minor functional repercussions. CCA was partially deteriorated in both cases. The reconstruction was done for an aesthetic purpose. Previous expansion was carried out with placement of a cutaneous expander at the level of the supraclavicular region. The time between the first and second procedures ranged from 14 weeks to 20 weeks. The size of the OCS flap varied from 24 to 27 cm in length and from 7 to 8 cm in width (Figure 2(b)). The prosthetic shell was not included when elevating the flap. Flap thinning was realized in one case. The flaps were transposed to the cervical region at an angle of 90° and 100°. The donor site was closed primarily. In both cases, a distal venous congestion took place in the first days following surgery and evolved well. Total healing was achieved after 22 to 26 days. Our results were assessed after 14 to 18 months. The functional result was satisfactory with normal and complete neck function. No recurrence of retraction was observed. However, there was not enough improvement in the reconstruction of the CCA with a “neck-collar” aspect in both cases. As for the donor site flap (supraclavicular), we described a wide hypertrophic scar (Figure 2(b)), leading to a medium aesthetic appearance.

## 4. Discussion

The skin of the anterior and lateral sides of the neck has a specific structure. It is thin enough to fit the contours of the neck allowing mobility and is thick enough to protect the underlying vital structures [1, 3, 14]. Reconstruction of the postburn cervical contractures is aimed at restoring the cervical-chin angle and the cutaneous elasticity and suppressing extrinsic retraction forces [1]. All repair procedures could be used from skin grafts to the free flap [15]. In general, local flaps are preferable over distant flaps.

Occipito-cervico skin flaps were found to be useful in the reconstruction of postburn cervical contractures causing moderate or major functional impact. These flaps are adapted to the reconstruction of the vertical part of the neck. However, they do not restore the cervical-chin angle, and they give a “neck-collar” appearance. Besides, several degreasing procedures could be needed. Thomas reported in 1980 the principle of flaps with the dermal vascular network. This concept was the basis of the thin skin flap called “superthin flap,” described by Hyakusoku and Gao in 1994 [2]. Three types of flaps were described for the reconstruction of postburn cervical contractures: the “occipito-cervico-pectoral” flap centered on the pectoral region [2, 8], the “occipito-cervico-shoulder” flap centered on the clavicular region, and the occipito-cervico-dorsal flap centered on the back region [2, 8, 11, 16]. Their blood supply is ensured by the descending cutaneous branch of the occipital artery [10, 13] that anastomoses with the ascending branch of the transverse cervical artery. Its vascular territory might not reach the distal part of the flap [10, 13] which can be thinned immediately. Kuran et al. [3] categorize thin skin flaps according to their thickness into thin (5-10 mm), superthin (2-5 mm), and ultrathin (<2 mm). In our series, degreasing was carried out in three cases. In the other two cases, we found that the flaps were already thin enough.

Yazar et al. [17] showed that the distal part of the flap thinned can survive as an inert skin graft. In fact, it is nourished by flap bed osmosis and plasma imbibition [17]. Indeed, after the elevation of a skin flap, an insufficiency of blood circulation in the capillaries of the dermal network settles and is expressed by a purplish color in the intermediate and distal parts of the skin pallet. The dilation of small vessels in response to hypoxia could be seen in a few days (3 to 4 days). During this period, the survival of these areas is ensured by the vascular supply of the underground.

Gao et al. [6] showed in a series of 21 cases of preexpanded flaps for face and neck reconstructions that venous congestion of the distal part of the thinned flap was not related to flap degreasing and that the surviving zone depends essentially on the relationship between the width of the cutaneous pedicle and of the flap.

The technique of “supercharged flap” [17] overcomes this vascular problem which is seen when the ratio pedicle width/width of the flap is greater than 0.5. This technique makes it possible to increase the vascular supply and subsequently the size of the flap that can reach 40 cm∗20 cm for the OCD flap [7, 16].

The occipito-cervico flaps provide a large skin pallet and a great arc of rotation. The OCS flap may reach the mediocervical line of the neck and lower third of the face [6]. The random local flaps are indicated for small defects after releasing the contracture, sometime with a prior expansion [1]. The deltopectoral flap leaves a visible scar, which could be reduced by prior expansion [12]. In our practice, it remains an operation of last resort. The supraclavicular flap is easily harvested, has a constant anatomy, and is not very mutilating. It offers a good alternative in the reconstruction of small to medium cervicofacial defects [11, 15]. However, its distal part, beyond the clavicular region, has a random vascularization that increases the risk of its necrosis [18].

The cervical-chin angle separates the horizontal and vertical parts of the neck. Its reconstruction was not satisfying in our study. A “neck-collar” appearance of the flap was associated, especially when the contracture is partial and lies between the vertical and the horizontal parts of the neck. Some authors advocate the use of a skin graft for the reconstruction of the horizontal part and flaps for the reconstruction of the vertical part [1], but this requires a prolonged immobilization to ensure the good maintenance of the graft.

The aesthetic appearance of the donor site depends on the width of the flap. Prior expansion (3 patients in our series) reduces the scarring by allowing first-line closure and avoiding skin grafts. The capsulotomy, in the second operation, does not compromise the vascularization of the skin pallet [19–21]. However, the main disadvantage of this technique is the requirement of two procedures. The process lasts about 17 weeks, including the two-setting surgery [6] (between 14 and 20 weeks in our series). The other drawbacks are the risk of infection, cutaneous necrosis, and perforation or externalization of the expander [2, 3, 6, 11, 22].

The low number of patients in our series did not allow us to do a comparison with the other surgical techniques practiced for the reconstruction of postburn cervical contractures.

## 5. Conclusion

Given the diversity of surgical techniques proposed in the literature and the variability of surgeons' experience according to cultural, ethnic, and material factors, it seems to us that a meta-analysis is necessary in order to release a guideline for the reconstruction of postburn cervical contractures. Otherwise, the use of occipito-cervico flaps is recommended in the repair of major burn sequelae affecting the horizontal anterior part of the neck.

## Figures and Tables

**Table 1:** Epidemiological and clinical characteristics of the patients.

Case	Age (year)	Sex (M/W)^∗^	Burn mechanism	Location	Extrinsic retraction	Classification of Vandenbussche		CCA^∗∗^
No. 1	29	M	Thermic	Pancervical	Yes	Major retractile neck	Major retractile neck	Deleted
No. 2	37	M	Thermic	Laterocervical	No	Segmental retractile neck	Medium retractile neck	Partially deleted
No. 3	21	M	Thermic	Laterocervical	No	Segmental retractile neck	Medium retractile neck	Partially deleted
No. 4	25	W	Thermic	Mediocervical	No	Segmental retractile neck	Minor retractile neck	Partially deleted
No. 5	14	M	Thermic	Mediocervical	No	Segmental retractile neck	Minor retractile neck	Partially deleted

^∗^M: man/W: woman; ^∗∗^CCA: cervical-chin angle.

**Table 2:** The characteristics of thin skin flaps.

Case	Flap type (OCS/OCD)^∗^	Prior expansion	Size (cm)	Pedicle width (cm)	Donor site	Thinning	Transposition (degree)	Distal congestion (cm)	Evolution	Healing (day)	CCA^∗∗^	Neck function
No. 1	OCD	No	30 × 8	5	Primary intention	Yes	110	4 × 3	Superficial epidermolysis	30	Neck-collar aspect	Restored
No. 2	OCD	No	25 × 8	4	Primary intention	No	110	3 × 8	Superficial epidermolysis	36	Neck-collar aspect	Restored
No. 3	OCD	No	23 × 6	4	Primary intention	Yes	110	2 × 6	Superficial epidermolysis	29	Neck-collar aspect	Restored
No. 4	OCS	Yes	27 × 7	4	Primary intention	No	90	9 × 4	Superficial epidermolysis	22	Neck-collar aspect	Restored
No. 5	OCS	Yes	24 × 8	4	Primary intention	Yes	100	12 × 8	Superficial epidermolysis	26	Neck-collar aspect	Restored

^∗^OCD: occipito-cervico-dorsal/OCS: occipito-cervico-shoulders; ^∗∗^CCA: cervical-chin angle.

## References

[1] Pradier J.-P., Duhamel P., Brachet M., Dantzer E., Vourey G., Bey E. (2011). Stratégie chirurgicale des brûlures du cou et de leurs séquelles. *Annales de Chirurgie Plastique Esthétique*.

[2] Hyakusoku H., Gao J. H. (1994). The “Super-thin” flap. *British Journal of Plastic Surgery*.

[3] Kuran I., Turan T., Sadikoglu B., Ozcan H. (1999). Treatment of a neck burn contracture with a super-thin occipito-cervico-dorsal flap: a case report. *Burns*.

[4] Orgill D. P., Ogawa R. (2013). Current methods of burn reconstruction. *Plastic and Reconstructive Surgery*.

[5] Frame J. D., Still J., Lakhel-LeCoadou A. (2004). Use of dermal regeneration template in contracture release procedures: a multicenter evaluation. *Plastic and Reconstructive Surgery*.

[6] Gao J.-H., Ogawa R., Hyakusoku H. (2007). Reconstruction of the face and neck scar contractures using staged transfer of expanded “Super-thin flaps”. *Burns*.

[7] Ogawa R., Hyakusoku H., Murakami M., Gao J.-H. (2004). Clinical and basic research on occipito-cervico-dorsal flaps: including a study of the anatomical territories of dorsal trunk vessels. *Plastic and Reconstructive Surgery*.

[8] Motamed S., Kalantar Hormozi A. J., Marzban S. (2003). Expanded occipito-cervico-pectoral flap for reconstruction of burned cervical contracture. *Burns*.

[9] Hassan S., Brooks P. (2014). Pre-expanded occipito-dorsal flap reconstruction for neck burns: a novel approach. *Burns & Trauma*.

[10] Tsukada S. (1980). Transfer of free skin grafts with a preserved subcutaneous vascular network. *Annals of Plastic Surgery*.

[11] Pallua N., Demir E. (2008). Postburn head and neck reconstruction in children with the fasciocutaneous supraclavicular artery island flap. *Annals of Plastic Surgery*.

[12] Bey E., Hautier A., Pradier J.-P., Duhamel P. (2009). Is the deltopectoral flap born again? Role in postburn head and neck reconstruction. *Burns*.

[13] Vandenbussche F., Vandevord J., Decoopman B., Decoulx P. (1978). 30 cases of cervical sequelae of burns: morphological types and special technical points. *Annales de Chirurgie Plastique*.

[14] Mimoun M., Kirsch J. M., Faivre J. M., Baux S. (1986). Rebuilding the cervico-mandibular angle--correcting a deformity of neck burns. *Burns*.

[15] Pallua N., Machens H. G., Rennekampff O., Becker M., Berger A. (1997). The fasciocutaneous supraclavicular artery island flap for releasing postburn mentosternal contractures. *Plastic and Reconstructive Surgery*.

[16] Minagawa T., Kimura C. (2008). Microvascularly augmented occipito-cervico-dorsal flap for a large soft tissue defect secondary to severe cervical abscess. *Journal of Plastic, Reconstructive & Aesthetic Surgery*.

[17] Yazar S., Zeki Guzel M., Aydın Y., Arslan H., Demır M. (2008). Demonstration of circulation haemodynamics in random pattern thinned skin flap (an experimental study). *Journal of Plastic, Reconstructive & Aesthetic Surgery*.

[18] Vinh V. Q., Van Anh T., Ogawa R., Hyakusoku H. (2009). Anatomical and clinical studies of the supraclavicular flap: analysis of 103 flaps used to reconstruct neck scar contractures. *Plastic and Reconstructive Surgery*.

[19] Dast S., Vaucher R., Rotari V. (2017). Les lambeaux cutanés minces dans la prise en charge des pertes de substance cutanée de la main et du membre supérieur. *Annales de Chirurgie Plastique Esthétique*.

[20] Patel P. A., Elhadi H. M., Kitzmiller W. J., Billmire D. A., Yakuboff K. P. (2014). Tissue expander complications in the pediatric burn patient: a 10-year follow-up. *Annals of Plastic Surgery*.

[21] Pribaz J. J., Fine N., Orgill D. P. (1999). Flap prefabrication in the head and neck: a 10-year experience. *Plastic and Reconstructive Surgery*.

[22] Hyakusoku H., Pennington D. G., Gao J. H. (1994). Microvascular augmentation of the super-thin occipito-cervico-dorsal flap. *British Journal of Plastic Surgery*.

